# Effectiveness of contralaterally controlled functional electrical stimulation vs. neuromuscular electrical stimulation for recovery of lower extremity function in patients with subacute stroke: A randomized controlled trial

**DOI:** 10.3389/fneur.2022.1010975

**Published:** 2022-12-08

**Authors:** Songhua Huang, Yuqian Zhang, Peile Liu, Yinglun Chen, Beiyao Gao, Chan Chen, Yulong Bai

**Affiliations:** Department of Rehabilitation Medicine, Huashan Hospital, Fudan University, Shanghai, China

**Keywords:** neuromuscular electrical stimulation, contralaterally controlled functional electrical stimulation, stroke, lower extremity motor function, ankle dorsiflexion

## Abstract

**Objective:**

This study aimed to compare the efficacy of contralaterally controlled functional electrical stimulation (CCFES) vs. neuromuscular electrical stimulation (NMES) for motor recovery of the lower extremity in patients with subacute stroke.

**Materials and methods:**

Seventy patients within 6 months post-stroke were randomly assigned to the CCFES group (*n* = 35) and the NMES group (*n* = 35). Both groups underwent routine rehabilitation plus 20-min electrical stimulation (CCFES or NMES) on ankle dorsiflexion muscles per day, 5 days a week, for 3 weeks. Ankle AROM (dorsiflexion), Fugl-Meyer assessment-lower extremity (FMA-LE), Barthel Index (BI), Functional Ambulation Category scale (FAC), 10-meter walking test, and surface electromyography (sEMG) were assessed at the baseline and at the end of the intervention.

**Result:**

Ten patients did not complete the study (five in CCFES and five in NMES), so only 60 patients were analyzed in the end. After the 3-week intervention, FMA-LE, BI, Ankle AROM (dorsiflexion), and FAC increased in both groups (*p* < 0.05). Patients in the CCFES group showed significantly greater improvements only in the measurement of Fugl-Meyer assessment-lower extremity compared with the NMES group after treatment (*p* < 0.05). The improvement in sEMG response of tibialis anterior by CCFES was greater than NMES (*p* < 0.05).

**Conclusion:**

Contralateral controlled functional electrical stimulation can effectively improve the motor function of the lower limbs better than conventional neuromuscular electrical stimulation in subacute patients after stroke, but the effect on improving the ability to walk, such as walking speed, was not good.

**Clinical trial registration:**

http://www.chictr.org.cn/, identifier: ChiCTR2100045423.

## Introduction

There are more than 2 million new stroke cases in China every year, and 70~80% of the patients lose their capability for independence due to many kinds of dysfunctions (1, 2). Of these, motor dysfunction can restrict patients' mobility functions and impair their independence, and thus has a serious impact on the quality of life of both patients and their families. Motor recovery is one of the main goals of stroke rehabilitation (3). For mobility function, lower limb motor function and walking ability are pivotal. Patients with stroke with walking problems often presented with poor dorsiflexion of the affected ankle, causing clearance impairment and circling gait. Strengthening the ankle dorsiflexion function of the lower extremity in the early phase of rehabilitation can not only effectively prevent ankle contractures, but also play an important role in restoring mobility, improving gait, and preventing falls (4).

Neuromuscular electrical stimulation (NMES) is an effective and conventional treatment for promoting the recovery of the lower extremity motor function in patients with stroke (5). During NMES treatment, low-frequency current pulses are applied to the muscles or motor nerves through surface electrodes to cause muscle contraction (6). NMES can help increase and maintain joint range of motion (ROM), prevent disuse muscle atrophy, and promote motor relearning (7). However, in most application scenarios, NMES triggers the movement in a passive form. The frequency and amplitude of electrical stimulation are pre-set and fixed during the whole training phase. For better functional recovery, the patient's subjective attempts are encouraged to be combined with neuromuscular electrical stimulation, thus “functionalizing” the NMES. The first report about how to “functionalize” neuromuscular electrical stimulation was published in 1961 by Liberson et al., who took the first trial of applying neuromuscular electrical stimulation to improve the ankle dorsiflexion function of patients with stroke (8). To further promote the functional gain of NMES, functional electrical stimulation (FES) was widely used. For decades, studies on FES are increasing, but the implementation of FES mostly requires patients to preserve certain motor functions. Those patients in the acute phase or who have severe dysfunctions are impossible to complete the application, and the effectiveness of FES implementation depends on the functional phase of the affected side (9, 10).

Contralaterally controlled functional electrical stimulation (CCFES) is a recently developed technique for promoting motor recovery of limbs after stroke. CCFES uses a joint angle sensor from the movement of the unaffected limb to trigger the stimulator to allow the affected limb to generate the same movement as the unaffected limb. Different from NMES, the movement of the affected limb can be controlled by the patients themselves, and the movement of the two sides is bilateral symmetrical during CCFES. Another unique advantage is that CCFES can be performed by combining with patients' subjective efforts even in the acute phase or severe motor dysfunction. The first article about CCFES was published in 2007 by Knutson et al. (11). Though more relevant studies have emerged since then, the findings on the application of CCFES equipment to improve the recovery of ankle dorsiflexion function after stroke are few. Moreover, a pilot study supported the feasibility and effectiveness of CCFES for ankle dorsiflexion (12). However, a later RCT generated no significant differences between groups in any of the outcome measures, suggesting CCFES was no better than cyclic NMES for ankle dorsiflexion (13). It is unknown whether the controversial results were due to the limitations of the treatment or the different recovery laws of the upper and lower limbs. In addition, since most of the previous studies utilized assessment scales to evaluate the changes in patients' mobility function between baseline and end of treatment, the final result could be inevitably biased by the evaluator's subjective judgment (14, 15). Therefore, based on the assessment scale in this study, surface electromyography (sEMG) was introduced and the effects of CCFES and NMES on the functional recovery of lower extremities in patients with stroke were quantified by muscle activation.

The aim of this study is to compare the efficacy of CCFES vs. NMES for recovery of the lower extremity in 6 months post-stroke by lower limb functional assessment and surface electromyography (sEMG) evaluation.

## Materials and methods

This study was designed as a parallel randomized controlled trial. The outcome assessments were evaluated by doctors who were blinded to the allocation. The Ethics Committee of Huashan Hospital, Fudan University approved the study protocol (the approval number was 2021-490). This study was registered in the Chinese Clinical Trial Registry (http://www.chictr.org.cn/) (No. ChiCTR2100045423).

### Subjects

Patients with lower limb motor dysfunction after stroke were recruited from the Department of Rehabilitation Medicine, Baoshan Branch of Huashan Hospital, Fudan University, from April 2021 to March 2022. All patients or their legally authorized representatives were informed about this study and provided written consent prior to the study.

Inclusion criteria were as follows: (1) diagnosis of a first-ever stroke with unilateral lesion confirmed by head CT or MRI scanning; (2) general condition with stabilized vital signs and normal consciousness; (3) score of mini-mental state examination ≥24; (4) aged between 30 and 80 years; (5) Brunnstrom recovery stage one to four for the affected lower limb; (6) 7 days to 6 months after stroke onset (16); and (7) volunteered for this study with signed informed consent.

Exclusion criteria were as follows: (1) reversible stroke; (2) severe visceral organ dysfunction (e.g., heart, lung, liver, or kidney dysfunction); (3) speech and hearing impairments; (4) history of mental disease and inability to cooperate with treatment and assessment; (5) cardiac pacemaker implanted; (6) unable to be followed up regularly, or unable to receive treatment in designated hospital at a specific time; and (7) lower extremity dysfunction due to other causes.

The administrative assistant of the study who did not participate in the treatment and assessment assigned the patients to either the NMES or the CCFES group using a random number table generated by computer and assigned to two groups in a 1:1 ratio by concealed sequentially numbered envelopes.

### Study protocol

Patients in both groups went through routine rehabilitation (1 h/day) for 5 days per week over a period of 3 weeks, including posture management (e.g., sitting, standing, and sit-to-stand), Bobath therapy, and proprioceptive neuromuscular facilitation therapy. Therapists who performed the routine rehabilitation were blinded to group allocation. CCFES or NMES was provided in addition to routine rehabilitation training. Accompanying diseases (e.g., coronary artery, hypertension, and diabetes) were treated with medicines.

In the CCFES group, a DC-L-500 contralaterally controlled functional electrical stimulator (Jiangsu NeuCognic Medical Co., Ltd, Jiangsu, China) was used to stimulate the tibialis anterior of the paretic side controlled by the non-paretic side. Subjects sat with the knees slightly flexed and the feet placed on an oblique board to keep the ankles in the neutral position with the relaxation of the lower limb. The surface electrodes were placed on the muscle belly of the tibialis anterior of the paretic side. The joint angle sensor for detecting non-paretic ankle dorsiflexion and triggering stimulation on the paretic tibialis anterior was worn on the dorsal surface of the non-paretic foot. Before stimulation, subjects were asked to voluntarily dorsiflex the non-paretic ankle to a certain angle according to the instruction (0, 20, and 15 degrees) and recorded by the joint angle sensor. The dorsiflexion of the paretic ankle was elicited by the electrical stimulation from the joint angle sensor when it detected the motion of the non-paretic ankle (at least a 15-degree dorsiflexion). The stimulation aims to generate 15 degrees of ankle dorsiflexion on the paretic side. The therapist would instruct the non-paretic ankle dorsiflexion and adjust the stimulating intensity ensuring to elicit the 15-degree ankle dorsiflexion on the paretic side without causing pain or any discomfort (a sensory sustainable range). The subjects were instructed to relax the paretic leg during the treatment. The waveform of stimulation was a biphasic rectangular wave with a frequency of 35 pps and a pulse width of 200 μs. The subjects were asked to maintain the non-paretic ankle dorsiflexion for 10 s so that the stimulation on the paretic side could last. Once the non-paretic ankle relaxed and went back to 0 degrees, the stimulation ceased. The interval of every motion and stimulation was set as 10 s. A 5-min practice session was initially performed before commencing the CCFES therapy to ensure that the subjects know how to participate in the treatments.

In the NMES group, a MyoNet-BOW neuromuscular electrical stimulator (Shanghai NCC Electronic Co., Ltd, Shanghai, China) delivered a biphasic rectangular wave with 35 pps and a pulse width of 200 μs. The subjects sat with the knees slightly flexed, and the feet placed on an oblique board to keep the ankles in the neutral position with the relaxation of the lower limb. The surface electrodes were placed on the muscle belly of the tibialis anterior on the paretic side. The subjects were instructed to relax the paretic leg during the treatment. The stimulation and relaxation time was set as 10:10 s. The stimulation intensity was adjusted to the level of tetanic contraction, which would elicit the 15-degree ankle dorsiflexion of the paretic ankle, without causing any pain sensation.

Both groups received the electrical stimulation 20 min/session, 1 session/day, 5 consecutive days/week, for 3 weeks.

### Outcome assessment

Two doctors who performed the functional evaluations were blinded to group allocation at baseline and after a 3-week intervention.

The primary outcome is the active range of motion of ankle dorsiflexion: The subjects sat with the knee 90°flexed and the heels placed on the ground and focussed on preventing ankle inversion and eversion. When measuring the range of motion of the ankle dorsiflexion, the goniometer center (“0” point) was placed over the intersection of the longitudinal axis of the fibula and the outer edge of the foot, which is about 2 cm below the lateral malleolus. The stationary goniometer arm was aligned parallel to the longitudinal axis of the fibula, and the mobile arm was placed parallel to the longitudinal axis of the fifth metatarsal bone. Then, the subjects were required to try their best to dorsiflex the ankle, repeat three times, and take the one with the largest value.

The secondary outcomes included the following.

#### Surface electromyography (sEMG)

A MyoMove-EOW apparatus (Shanghai NCC Electronic Co., Ltd, Shanghai, China) was used to collect the sEMG signals of the tibialis anterior and gastrocnemius lateralis on both sides, which were recorded during active ankle dorsiflexion. The signal was amplified and band-pass-filtered (5–500 Hz) prior to sampling. The subjects were trained before signal collection to understand the whole procedure. Before starting, the posture of the subject is supine, and the leg is supported just above the ankle joint. The surface electrodes need to be placed at one-third on the line between the tip of the fibula and the tip of the medial malleolus. During the collection, the subjects were required to try their best to dorsiflex the ankle and maintain for about 3 s and then relax for 5 s, repeating three times. The signals were recorded and generated automatically to the root mean square (RMS) values by the software installed with the surface electromyography apparatus. The RMS of the paretic tibialis anterior was standardized by calculating the sEMG signal ratio in percentage, a ratio of RMS of the paretic side/the non-paretic side.

#### Barthel index

Ten items were used to evaluate the ability of daily living. The total score is 100, including feeding, toilet use, fecal and urinary incontinence, dressing and undressing, grooming, bathing, walking, climbing stairs, and transfer (e.g., from chair to bed). The higher the score, the better the level of activities of daily living.

#### The Fugl-Meyer motor assessment of lower extremity (FMA/motor-LE)

The Fugl-Meyer motor assessment of the lower extremity evaluates the tendon reflexes and the performance of given tasks involving the hip, the knees, and the ankles. Each scoring item was graded on a three-point scale from 0 to 2, except for the reflex activity which has only two points, scoring 0 or 2. Scoring 0 means no reflex can be elicited or the volitional movement cannot be performed at all. Scoring 1 means the motion can be performed partially. Scoring 2 means the motion can be performed fully. The maximum score of the lower extremity Fugl-Meyer motor assessment is 34.

#### The functional ambulation category (FAC)

The six-point scale assesses ambulation status by determining how much human support the patient requires when walking, regardless of whether they use a personal assistive device or not. The FAC is a functional walking test that evaluates ambulation ability. The FAC does not evaluate endurance, as the patient is only required to walk approximately 10 feet.

#### The timed 10-meter walking test

Two lines were drawn on the ground at a distance of 10 meters. The patient stood at the beginning line and was asked to walk at normal speed until reaching the ending line. The walking time taken by patients was recorded using a stopwatch. Three consecutive trials were conducted, and the mean time was calculated.

### Statistical analysis

An SPSS 24.0 version (Statistical Package for Fudan University) was used for statistical analysis. For baseline demographic and clinical characteristic comparability, a chi-square test was used for categorical variables. The continuous data were checked for normality using the Shapiro–Wilk test; continuous data were presented as means with standardized deviation (SD) when data conformed to normal distribution; otherwise, the median (IQR) was applied. Categorical data were represented as composition ratio. Paired *t*-tests were used for intragroup comparisons, while independent samples *t*-tests were used for comparisons between groups. For non-normally distributed data or data lacking homogeneity of variance, the Wilcoxon rank-sum test (Mann–Whitney U-test) was used to compare paired and between-group samples. The significance level was set at 0.05.

The sample size was calculated according to the data from Knutson et al. (13), using ankle dorsiflexion AROM as the main evaluation index. To achieve 90% power with a level of significance of 0.05, a minimum sample size of 52 patients (26 per group) was needed to detect statistical significance for a between-group difference in ankle dorsiflexion AROM. Assuming a 20% dropout rate, the minimum number of enrolled patients was determined to be 66 (33 per group). PASS 15.0 was used for sample size calculation.

## Results

**Figure 1:**
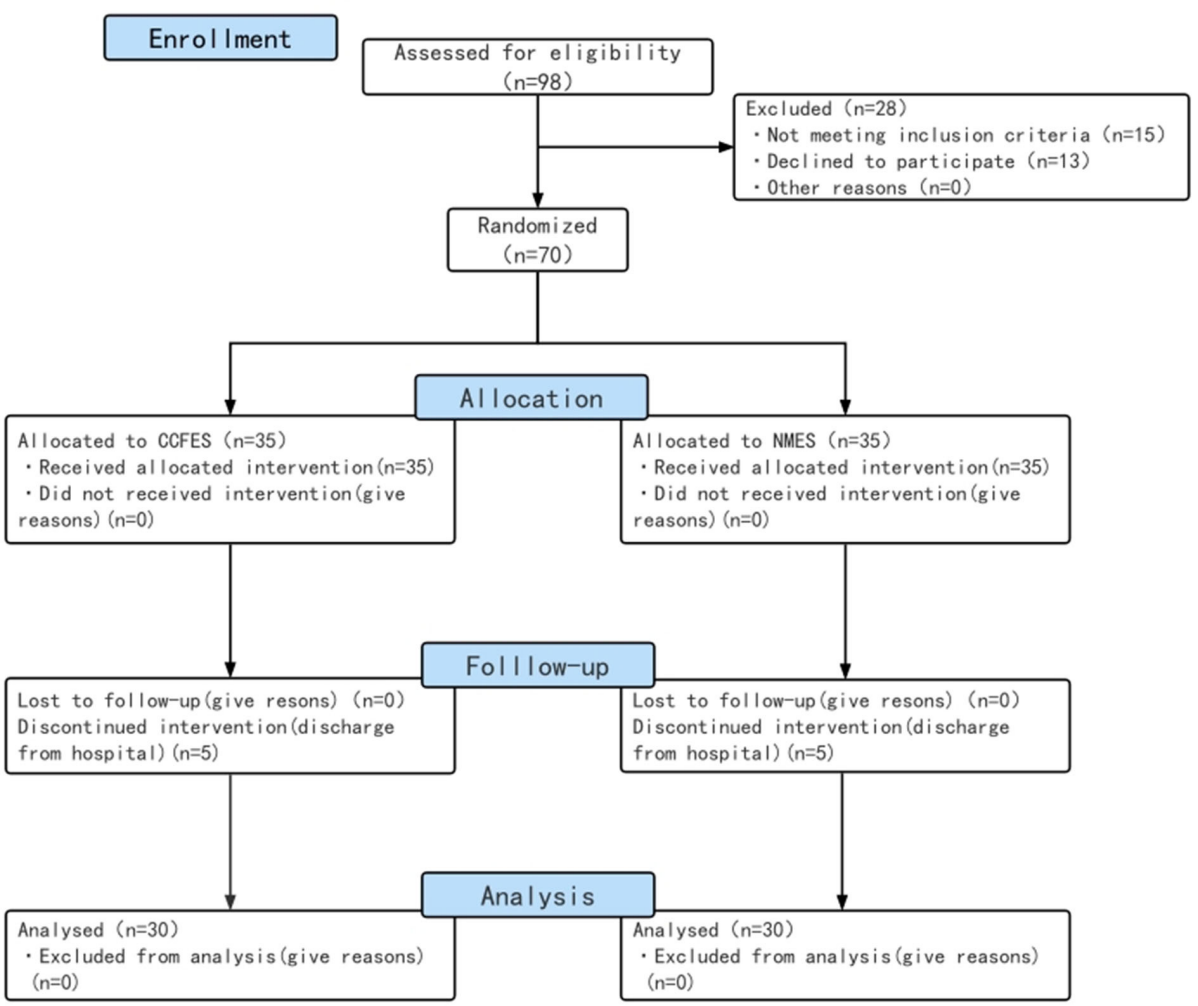
Study flow diagram. CCFES, contralaterally controlled functional electrical stimulation; NMES, neuromuscular electrical stimulation.

A total of 70 eligible patients were enrolled and randomly assigned into the NMES group (*n* = 35) and the CCFES group (*n* = 35) (Figure 1). There were no significant differences between groups in gender, type of stroke, side of affected hemisphere, and Brunnstrom recovery stage according to the chi-square test (*p* > 0.05), nor in age and course of disease according to the Mann–Whitney U-test (*p* > 0.05) (Table 1).

**Table 1:** Baseline demographics and clinical characteristics.

**Characteristic**	**CCFES (*n* = 30)**	**NMES (*n* = 30)**	***P*-value**
Age (years)	58 (49.25, 63)	59 (51.25, 67.5)	0.245
Course of diseases (days since stroke)	30 (22, 77)	37 (22, 97.5)	0.534
**Gender**			0.999
Male	21 (70%)	21 (70%)	
Female	9 (30%)	9 (30%)	
**Type of stroke**			0.284
Ischemic	21 (70%)	17 (57%)	
Hemorrhagic	9 (30%)	13 (43%)	
**Hemisphere affected**			0.302
Left	13 (43%)	17 (57%)	
Right	17 (57%)	13 (43%)	
**Brunnstrom recovery stage**			0.774
I-III (lower limb)	22 (73%)	21 (70%)	
IV (lower limb)	8 (27%)	9 (30%)	
Ankle dorsiflexion AROM	0 (0, 5)	0 (0, 10)	0.665
FMA-LE	16.53 ± 7.06	13.83 ± 7.17	0.147
Barthel Index	52.17 ± 19.77	51.00 ± 19.09	0.817
10-meter walking test	81.53 ± 42.37	49.07 ± 29.58	0.078
FAC	0 (0, 3)	0 (0, 2.25)	0.846
RMS of paretic TA	0.17 (0.04, 0.27)	0.07 (0.02, 0.39)	0.554

Results are presented as mean ± SD for normally distributed continuous variables, as median (interquartile range) for non-normally distributed continuous variables, and as number (percentage) for categorical data.

FAC, Functional Ambulation Category scale; FMA-LE, Fugl-Meyer assessment-lower extremity; TA, tibialis anterior.

After the 3-week intervention, Ankle AROM (dorsiflexion), FMA-LE, BI, and FAC increased in both groups (*p* < 0.05) (Table 2). The patients in the CCFES group showed significantly greater improvements only in FMA-LE compared with the patients in the NMES group after treatment (*p* < 0.05) (Table 2). For the sEMG evaluation, the improvement of RMS of the tibialis anterior in the CCFES group was greater than that in the NMES group (*p* < 0.05) (Table 3).

**Table 2:** Comparison of functional assessment and sEMG in two groups after treatment.

	**CCFES (*n* = 30) end-of-intervention**	**NMES (*n* = 30) end-of-intervention**	**Z**	***P*-value**
Ankle dorsiflexion AROM	4 (0, 15)*	2.5 (0, 10)*	−0.415	0.678
FMA-LE	22.5 (18.75, 25)*	16 (11.75, 21.25)*	−2.354	0.019^∧^
Barthel Index	65 (50, 75)*	57.5 (43.75, 76.25)*	−0.913	0.361
FAC	2.5 (0, 4)*	1 (0, 3.25)*	−1.055	0.292
10-meter walking test	54 (37.5, 88.2)	45.6 (22.93, 76.8)	−0.888	0.375
RMS of paretic TA	0.23 (0.14, 0.5)*	0.19 (0.06, 0.43)	−1.390	0.165

The data of the 10 meters walking test were from patients who could complete the assessment of walking both at the time of enrollment and end of intervention.

FAC, Functional Ambulation Category scale; FMA-LE, Fugl-Meyer assessment-lower extremity; TA, tibialis anterior.

^*^P < 0.05 indicates statistically significant differences in intragroup.

^∧^P < 0.05 indicates statistically significant differences between groups.

**Table 3:** Changes from baseline to end-of-intervention in functional assessments and sEMG.

	**CCFES (*n* = 30)**	**NMES (*n* = 30)**	**Z / t**	***P*-value**
Ankle dorsiflexion AROM	0 (0, 6.5)	0 (0, 5)	−1.139	0.255
FMA-LE	4.5 (0, 7.25)	3 (0, 5.25)	−0.868	0.385
Barthel index	10 (5, 15)	5 (0, 15)	−1.248	0.212
FAC	0 (0, 1.25)	0 (0, 1)	−0.802	0.422
RMS of paretic TA	0.1 (0.03, 0.19)	0.04 (−0.05, 0.16)	−1.996	0.046^∧^
10-meter walking test	−12.3 ± 16.34	−0.27 ± 6.88	−2.036	0.059

The data of the 10 meters walking test were from patients who could complete the assessment of walking both at the time of enrollment and end of intervention.

FAC, Functional Ambulation Category scale; FMA-LE, Fugl-Meyer assessment-lower extremity; TA, tibialis anterior.

^∧^P < 0.05 indicates statistically significant changes from baseline to end-of-intervention.

A FMA-LE motor subcategory analysis showed that flexor synergy, extensor synergy, movement combining synergy, movement out of synergy, and coordination/speed improved significantly in both groups (*p* < 0.05) (Table 4). Compared with the NMES group after treatment, patients in the CCFES group showed significantly greater improvements in extensor synergy, movement combining synergy, and movement out of synergy (*p* < 0.05) (Table 4).

**Table 4:** Comparison of FMA-LE motor subcategories in two groups after treatment.

**FMA-LE (motor)**	**CCFES (*n* = 30) end-of-intervention**	**NMES (*n* = 30) end-of-intervention**	**Z**	***P*-value**
Reflex activity	4 (4, 4)	4 (4, 4)	1.062	0.288
Flexor synergy	3.5 (2, 5) *	2.5 (1.5, 4.25) *	1.76	0.078
Extensor synergy	6 (4, 7) *	4 (2, 5) *	2.817	0.005^∧^
Movement combining synergy	2 (1, 3) *	1 (0, 2) *	2.384	0.017^∧^
Movement out of synergy	2 (0, 2) *	0.5 (0, 1) *	2.396	0.017^∧^
Normal reflexes	2 (1, 2) *	1.5 (0, 2)	0.831	0.406
Coordination/speed	4 (3, 5) *	5 (2, 6) *	1.06	0.289

^*^P < 0.05 indicates statistically significant differences in intragroup.

^∧^P < 0.05 indicates statistically significant differences between groups.

No adverse events were reported during the intervention and follow-up in any of the groups.

## Discussion

The application of CCFES to treat ankle dorsiflexion dysfunction after stroke was first reported by Knutson et al. (12). This pilot study (*n* = 3, time since stroke: >6 months) supported the feasibility and effectiveness of CCFES to improve ankle dorsiflexion. However, a later RCT (CCFES vs. cyclic NMES, *n* = 26, time since stroke: ≥6 months) (13) generated no significant differences between groups in any of the outcome measures, suggesting CCFES was not superior to cyclic NMES for ankle dorsiflexion in chronic stroke. The authors further discussed and attributed to the default inter-leg coordination in anti-phase, while CCFES stimulated bilateral legs simultaneously. Further investigation on the anti-phase CCFES might generate more benefits. To observe the effectiveness of CCFES in different recovery stages of lower limb dysfunction after stroke, the target group of this study was patients with stroke in the subacute phase (CCFES VS. NMES, n=60, 7 days < time since stroke < 6 months, the average time since stroke is about 50 days in average), and the results supported the conclusion that CCFES is superior to NMES in the treatment of lower extremity motor dysfunction in the subacute phase after stroke, which may indicate that patients with stroke in the subacute phase tend to benefit more from CCFES than those in chronic phase.

Patients in the CCFES group did not show a significantly higher improvement in the assessment of ankle AROM (dorsiflexion) than that in the NMES group (*P* > 0.05), but patients in the CCFES group gained significantly higher FMA-LE compared with the NMES group after treatment. With further analysis, the subcategories of extensor synergy, movement combining synergy, and movement out of synergy in FMA-LE differed significantly between the two groups after treatment. For the assessment of these subcategories, ankle dorsiflexion is critical for scoring. This implies that CCFES may have contributed to better performance in motion assessment. For the sEMG evaluation, the improvement of RMS of the tibialis anterior in the CCFES group was significantly higher than that in the NMES group (*P* < 0.05). It may indicate that NMES can effectively improve the recruitment of muscle fibers (17), but CCFES is more effective in activating the number and synchronization of motor units during muscle contraction, which is consistent with the previous results of Huang et al. (18) in the upper extremity study. After the 3-week intervention, the functional scores of the two groups, except for the 10-meter walking test, were significantly improved in both groups (*P* < 0.05). Since not all patients in the initial enrollment could be assessed for walking, the data source of the 10-meter walking test was from patients who could complete the assessment of walking at both the time of enrollment and end of intervention (at the time of enrollment: CCFES group *n* = 9, NMES group *n* = 9; end of intervention: CCFES group *n* = 15, NMES group *n* = 10). Stroke recovery stages can be divided into hyperacute phase (0–24 h), acute phase (1–7 days), early subacute (7 days to 3 months), late subacute (3–6 months), and chronic phase (>6 months) (19). Different stroke stages correspond to different effective treatments and suitable strategies (20). Both the stroke stage and functional stage should be taken into consideration for a more accurate treatment strategy design. The statistical result of the 10-meter walking test in these two groups may suggest that the role of CCFES in different functional stages of patients is not the same. For patients who are unable to walk, CCFES can increase the activation number and synchronization rate of motor units compared to NMES, thus helping patients resume their walking ability. On the contrary, for patients whose muscles have recovered to a certain extent and sustain some mobility function, their self-motivated involvement in mobility activities is also increasing. Thus, CCFES no longer shows advantages compared to NMES in improving walking speed, which is used as a metric for measuring patients' walking performance. This indicates the possible reason for the observation of no significant improvement in the walking assessment. In addition, although CCFES is a functional electrical stimulation based on NMES, the treatment did not simulate walking, since the patients only completed the ankle dorsiflexion movement during treatment, which is different from the treatment of walking practice with the assistance of FES. Therefore, the result also suggested that previous analysis may not be true, in which they argue the reasons for the ineffectiveness of CCFES in the treatment of the lower extremity in chronic stroke were the default inter-leg coordination in anti-phase, while CCFES stimulated bilateral legs simultaneously. There are many factors affecting gait and determining walking ability. More studies need to be conducted to draw reliable conclusions.

CCFES is an improved form of NMES. NMES is widely used in stroke rehabilitation to facilitate motor relearning, ameliorate spasticity, prevent muscle atrophy and disuse osteoporosis, and preserve muscle protein synthesis (21, 22). CCFES has potential advantages that NMES does not have.

First, the CCFES exercise is an “intention-driven movement.” The objective muscle response is controlled by the participant's subjective exertion. Thus, the CCFES exercise needs high cognitive involvement. According to the “Hebbian plasticity,” “neurons that fire together, wire together” (23). The repetitive synchronized activation of central and peripheral neural pathways can promote neural reorganization.

Second, the CCFES exercise creates an illusion that the motor control of the affected limb is restored. This illusion can prevent or reverse learned non-use according to “mirror therapy theory” (24).

Last, CCFES is a kind of bilateral symmetric movement exercise. Interhemispheric inhibition (IHI) exists in the central neural system. After a stroke, the imbalanced IHI interferes with motor recovery. The theoretical model developed by Mudie and Matyas (25) suggests that bilateral symmetric movement can promote interhemispheric disinhibition and allow the ipsilesional hemisphere to share a “template of motor network recruitment” from the contralesional hemisphere. What is more, a recent crossover study (26) showed that CCFES (bilateral symmetric movement) reduced IHI and maintained ipsilesional output when compared with NMES (unilateral-based therapy).

Future studies should focus on measuring the effect of CCFES on central plasticity through functional magnetic resonance imaging (fMRI) and functional near-infrared spectroscopy (fNIRS). These assessments are intended to evaluate the central plasticity of the subject and to provide mechanism evidence for the discussion of relevant neuroplasticity mechanisms mentioned above.

There are several limitations to this study. First, the patients enrolled in this trial were between 7 days and 6 months after stroke. It is uncertain whether the results were affected by natural recovery, especially for those in early and late subacute stages. Second, the trial included patients with different Brunnstrom recovery stages of the lower limb, which may result in difficulty in evaluating the optimal effect of CCFES. Finally, the stimulation profile in this trial did not include the consideration of voluntary NMES on the unaffected side. This might ignore the effect of voluntarily controlled stimulation when comparing two stimulations.

## Conclusion

CCFES can effectively improve the motor function of the lower limbs better than conventional NMES in subacute patients after stroke, but the effect on improving the ability to walk, such as walking speed, was not good.

## Data availability statement

The raw data supporting the conclusions of this article will be made available by the authors, without undue reservation.

## Ethics statement

The studies involving human participants were reviewed and approved by the Ethics Committee of Huashan Hospital, Fudan University. The patients/participants provided their written informed consent to participate in this study. Written informed consent was obtained from the individual(s) for the publication of any potentially identifiable images or data included in this article.

## Author contributions

YZ, PL, YC, BG, CC, and YB participated in the study, including protocol design, experimental practice, data analysis, manuscript preparation, and approved the submitted version.
